# Dynamics of intracranial and peripheral plasma Syndecan‐1 after ischemic stroke with large vessel occlusion

**DOI:** 10.1111/cns.13898

**Published:** 2022-07-04

**Authors:** Tengkun Yin, Jiheng Hao, Qunlong Jiang, Xin Xu, Bin Xu, Hang Lv, Weidong Liu, Yilei Xiao, Liqun Jiao, Jiyue Wang, Liyong Zhang

**Affiliations:** ^1^ From the Department of Neurosurgery Liaocheng People's Hospital Liaocheng China; ^2^ Department of Neurosurgery Xuanwu Hospital, Capital Medical University Beijing China; ^3^ School of Clinical Medicine Weifang Medical College Weifang China

**Keywords:** acute ischemic stroke, glycocalyx, ischemia–reperfusion injury, large vessel occlusion, Syndecan‐1

Dear Editors,

1

Endothelial Syndecan‐1 is a principal constituent of glycocalyx which is now considered a cornerstone in ischemia–reperfusion‐related endothelial dysfunction as it participates in microvascular reactivity, endothelium interaction with blood constituents, and vascular permeability.^1^ Numerous pathologies such as trauma, inflammation, stroke, and ischemia–reperfusion injury can lead to increased shedding of Syndecan‐1.^1^, ^2^ The shedding of Syndecan‐1 and the temporal pattern of its release may represent important biomarkers serving as early‐stage predictors of organ damage. In this study, intracranial and peripheral plasma were collected in order to determine changes in Syndecan‐1 shedding, in a longitudinal large vessel occlusion (LVO) cohort.

This study was approved by the medical ethics committee (grant number, 202102037). Plasma samples were collected from eight healthy individuals (HI) aged 50–80 years, and from 24 patients with acute ischemic stroke due to middle cerebral artery occlusion (within 24 h). All participants were briefed and provided signatures of informed consent. All patients were being treated with thrombectomy with retrievable stent, were receiving standard medical treatment and, if appropriate, venous thrombolysis prior to thrombectomy. All patients achieved successful reperfusion (modified Thrombolysis in Cerebral Infarction [mTICI] score ≥2b). Exclusion criteria included: inflammatory diseases, and cancer; cytostatic/immunosuppressive therapy; and cerebral, heart, eye, or peripheral infarcts within 3 months. Clinical data collected include stroke severity, time from onset to groin puncture and successful reperfusion, with/without intravenous thrombolysis, the number of stentriever passes, and neurological functional outcome. Stroke severity was estimated using the National Institutes of Health Stroke Scale (NIHSS). Neurological functional outcome was estimated using the modified Rankin Scale (mRS) at 90 days.

Intracranial blood was aspirated from the offending vessel distal from the occlusion site during operation using a microcatheter^3^; peripheral blood was collected from all patients before groin puncture, and at 1 h, and 1, 3, and 7 days after successful reperfusion. All plasmas were stored at −80°C for subsequent analysis.

The plasma‐soluble Syndecan‐1 level was measured using ELISA kits according to the manufacturer's instructions (Abcam, Cambridge, MA, USA, Cat No. ab46506).

Data sets were tested for normality (Shapiro–Wilk). Non‐normal data sets were log‐transformed and tested again. Data sets that remained non‐normal were subject to nonparametric statistical tests. Parametric data were processed using the One‐Way ANOVA test, and non‐parametric data with the Kruskal–Wallis test. For Syndecan‐1, 1 h after reperfusion appeared to be the most dynamic sampling time. Therefore, we determined whether the variables (baseline NIHSS, the time from onset to groin puncture or successful reperfusion, with/without intravenous thrombolysis, number of stentriever passes) were predictive of the level of Syndecan‐1, 1 h after reperfusion. Data sets were tested using linear regression modeling. Correlations were tested for normality, and the Spearman coefficients were calculated. *p* values of <0.05 (SPSS 22.0) indicate significant differences.

Among the patients, the median stroke severity was moderate (baseline NIHSS 15, range 7–32). The median of 90‐day mRS was 2 (range 0–6). One patient died within 90 days. The percentage of males in the patient cohort was higher than that of the healthy group. There was no significant correlation between the Syndecan‐1 and gender at each timepoint, with or without LVO. It may therefore be argued that the level of plasma soluble Syndecan‐1 does not reflect the patient's gender. Nor was the level found to vary with age. Further, no significant correlations were found between Syndecan‐1, and (i) baseline NIHSS, (ii) the time from onset to groin puncture or successful reperfusion, (iii) number of stentriever passes, or (iv) 90‐day mRS at each timepoint.

Syndecan‐1 responded to ischemia–reperfusion injury in a diverse manner (Figure 1). In the hyper‐acute phase of LVO, the level of peripheral Syndecan‐1 was similar to that in HI. Within the intracranial plasma, the level of Syndecan‐1 increased significantly. Within 1 h after successful reperfusion, the level of Syndecan‐1 had increased sharply. At 1 day after reperfusion, it was found to have decreased substantially. Thereafter, the level of Syndecan‐1 gradually increased at 3 and 7 days after reperfusion.

**FIGURE 1:**
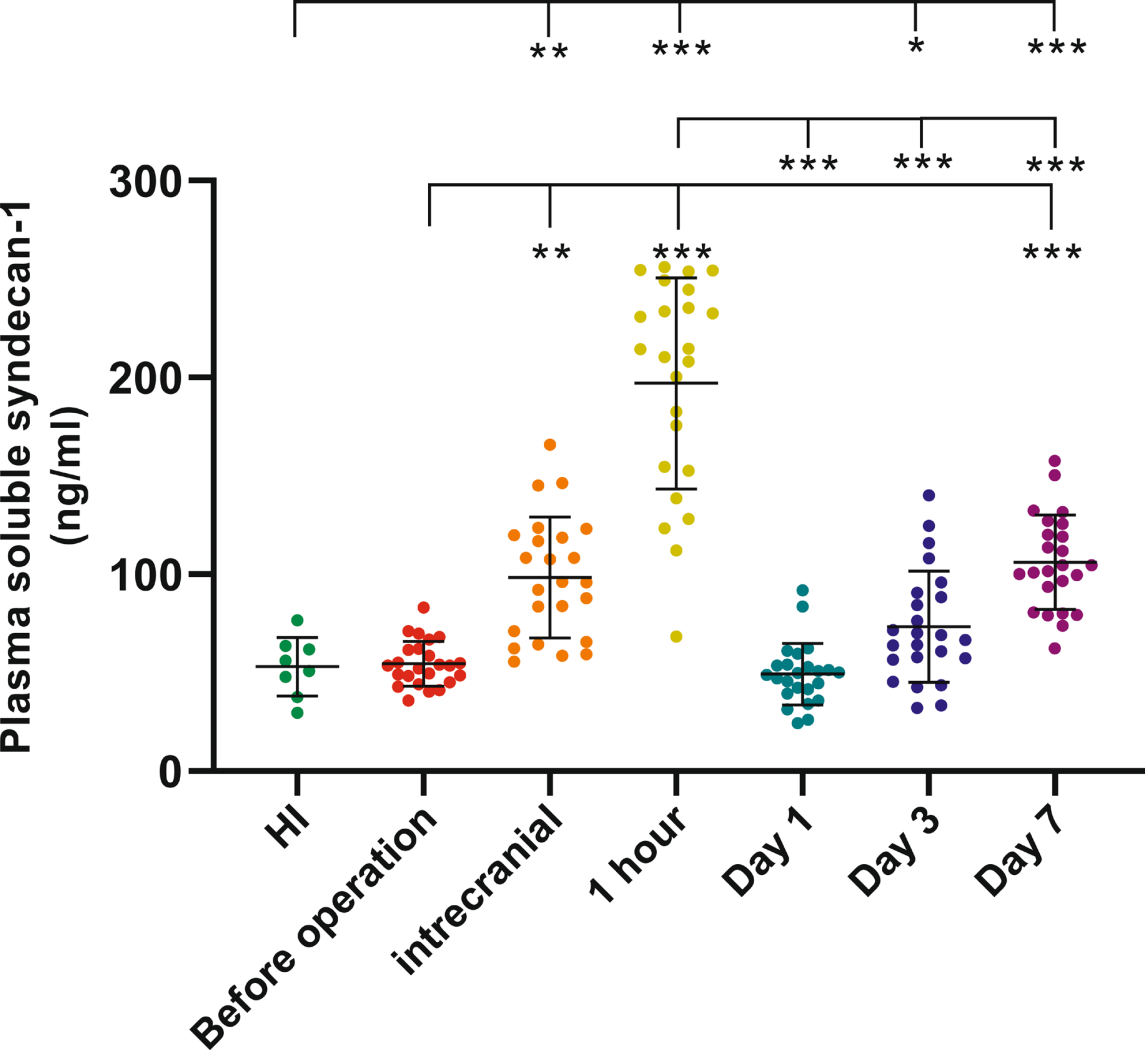
Syndecan‐1 detected in the plasma of healthy individuals (HI) and longitudinally after large vessel occlusion (LVO). Plasmas from HI (*N* = 8) and patients (*N* = 24) before operation, intraoperative(intracranial), and 1 h, 1 day, 3 days, and 7 days after recanalization after LVO were immunoassay for Syndecan‐1 marker. Data are presented as mean ± SD. *,**,***, represent *p* < 0.05, 0.01, and 0.001, respectively

Patients who underwent intravenous thrombolysis (*n* = 10) prior to thrombectomy showed lower levels of Syndecan‐1 both in the intracranial and in the peripheral plasma at 1 h after revascularization, compared with those who did not (*n* = 14) (*p* = 0.001).

In this article, we provide unique data showing that plasma soluble Syndecan‐1 displays a dynamic and complex signature following acute LVO. During the hyper‐acute period after stroke, there occurs a significant elevation of the level of Syndecan‐1 in the intracranial blood distal from the occlusion site, suggesting that Syndecan‐1 shedding initiates already during vascular occlusion.

More interestingly, at subsequent time points, we observed a biphasic pattern of soluble Syndecan‐1 elevation: shortly after recanalization, and 3–7 days after recanalization. This finding may indicate that Syndecan‐1 shedding is associated with increased blood–brain barrier permeability. At first, previous studies have confirmed that glycocalyx degradation is closely related to the impairment of vascular permeability.^4^ Second, the dynamic characteristics of Syndecan‐1 are similar to the currently known biphasic opening of the blood–brain barrier.^5^, ^6^ Accordingly, the protective therapy of Syndecan‐1 may become a new target for preventing or treating ischemia–reperfusion injury.

In addition, evidence has shown that the degree of recanalization by thrombectomy in LVO may be important for blood–brain barrier damage and permeability,^7^, ^8^ and, perhaps, the shedding of syndecan‐1 from vascular endothelial cells. Unfortunately, since all cases in this study achieved successful reperfusion (mTICI ≥2b), the relationship between the level of syndecan‐1 and recanalization by thrombectomy would not be substantiated in this investigation.

The significant association between venous thrombolysis and the Syndecan‐1 level may constitute additional evidence of the beneficial effect of venous thrombolysis for acute ischemic stroke in terms of glycocalyx protection.

The limited sample size is a significant drawback of this study; additional research studies with bigger sample sizes are necessary to assess the clinical importance of plasma soluble syndecan‐1.

In conclusion, this study provides the first direct evidence showing that the level of plasma Syndecan‐1 displays dynamic and complex changes following LVO. Our findings support experimental evidence showing that Syndecan‐1 may contribute to brain ischemia–reperfusion injury and blood–brain barrier damage, and indicate that Syndecan‐1 shedding occurs during vascular occlusion.

## AUTHOR CONTRIBUTIONS

Tengkun Yin, Jiheng Hao,Xin Xu, Liyong Zhang, and Liqun Jiao designed the study. Tengkun Yin, Jiheng Hao, Hang Lv, and Bin Xu drafted the first version of the manuscript and collected data. Bin Xu, Hang Lv, Yilei Xiao, Jiyue Wang, Liyong Zhang, Weidong Liu, and Liqun Jiao collected the data and made critical revisions to the manuscript. Tengkun Yin analyzed and interpreted the data.

## Funding information

This study was supported by the Clinical research team of Liaocheng People’s Hospital (Dr Wang), the Taishan Scholar Project of Shandong Province(grant number: tsqn202103200, Dr Xiao) and the Medical and Health Science and Technology Development Project of Shandong Province (grant number: 202104040390, Dr Yin).

## CONFLICT OF INTEREST

The authors report no competing interests.

## PATIENT CONSENT STATEMENT

All patients or their legal guardians provided their written informed consent.

## Data Availability

The data that support the findings of this study are available from the corresponding author on reasonable request.

## References

[1] Abassi Z , Armaly Z , Heyman SN . Glycocalyx degradation in ischemia‐reperfusion injury. Am J Pathol. 2020;190:752‐767. 32035883 10.1016/j.ajpath.2019.08.019

[2] Teixeira FCOB , Götte M . Involvement of Syndecan‐1 and Heparanase in cancer and inflammation. Adv Exp Med Biol. 2020;1221:97‐135. 32274708 10.1007/978-3-030-34521-1_4

[3] Kollikowski AM , Schuhmann MK , Nieswandt B , Müllges W , Stoll G , Pham M . Local leukocyte invasion during hyperacute human ischemic stroke. Ann Neurol. 2020;87:466‐479. 31899551 10.1002/ana.25665

[4] Nian K , Harding IC , Herman IM , Ebong EE . Blood‐brain barrier damage in ischemic stroke and its regulation by endothelial Mechanotransduction. Front Physiol. 2020;11:605398. 33424628 10.3389/fphys.2020.605398 PMC7793645

[5] Pillai DR , Dittmar MS , Baldaranov D , et al. Cerebral ischemia—reperfusion injury in rats—a 3 T MRI study on biphasic blood–brain barrier opening and the dynamics of edema formation. J Cereb Blood Flow Metab. 2009;29:1846‐1855. 19654585 10.1038/jcbfm.2009.106 PMC2848453

[6] Kuroiwa T , Ting P , Martinez H , Klatzo I . The biphasic opening of the blood‐brain barrier to proteins following temporary middle cerebral artery occlusion. Acta Neuropathol. 1985;68:122‐129. 3907257 10.1007/BF00688633

[7] Seners P , Turc G , Lion S , et al. Relationships between brain perfusion and early recanalization after intravenous thrombolysis for acute stroke with large vessel occlusion. J Cereb Blood Flow Metab. 2020;40:667‐677. 30890074 10.1177/0271678X19836288 PMC7026851

[8] Broocks G , Flottmann F , Hanning U , et al. Impact of endovascular recanalization on quantitative lesion water uptake in ischemic anterior circulation strokes. J Cereb Blood Flow Metab. 2020;40:437‐445. 30628850 10.1177/0271678X18823601 PMC7370621

